# Ion channels in EEG: isolating channel dysfunction in NMDA receptor antibody encephalitis

**DOI:** 10.1093/brain/awy107

**Published:** 2018-04-30

**Authors:** Mkael Symmonds, Catherine H Moran, M Isabel Leite, Camilla Buckley, Sarosh R Irani, Klaas Enno Stephan, Karl J Friston, Rosalyn J Moran

**Affiliations:** 1 Division of Clinical Neurology, John Radcliffe Hospital, Oxford, UK; 2 Department of Clinical Neurophysiology, John Radcliffe Hospital, Oxford, UK; 3 Epilepsy Research Group, Nuffield Department of Clinical Neurosciences, Oxford University, John Radcliffe Hospital, Oxford, Oxford, UK; 4 Department of Neurological Surgery, Beaumont Hospital, Dublin, Ireland; 5 Autoimmune Neurology Group, Nuffield Department of Clinical Neurosciences, Oxford University, John Radcliffe Hospital, Oxford, Oxford, UK; 6 Translational Neuromodeling Unit, Institute for Biomedical Engineering, University of Zurich and ETH Zurich, 6 Wilfriedstrasse, Zurich, Switzerland; 7 Wellcome Trust Centre for Neuroimaging, University College London, 12 Queen Square, London, UK; 8 Department of Engineering Mathematics, Merchant Venturers School of Engineering, University of Bristol, 75 Woodland Rd, Bristol, UK; 9 Department of Neuroimaging, Institute of Psychiatry, Psychology and Neuroscience, King's College London, London, UK

**Keywords:** NMDA receptor antibody encephalitis, EEG, encephalopathy, dynamic causal modelling

## Abstract

Neurological and psychiatric practice frequently lack diagnostic probes that can assess mechanisms of neuronal communication non-invasively in humans. In *N*-methyl-d-aspartate (NMDA) receptor antibody encephalitis, functional molecular assays are particularly important given the presence of NMDA antibodies in healthy populations, the multifarious symptomology and the lack of radiological signs. Recent advances in biophysical modelling techniques suggest that inferring cellular-level properties of neural circuits from macroscopic measures of brain activity is possible. Here, we estimated receptor function from EEG in patients with NMDA receptor antibody encephalitis (*n = *29) as well as from encephalopathic and neurological patient controls (*n = *36). We show that the autoimmune patients exhibit distinct fronto-parietal network changes from which ion channel estimates can be obtained using a microcircuit model. Specifically, a dynamic causal model of EEG data applied to spontaneous brain responses identifies a selective deficit in signalling at NMDA receptors in patients with NMDA receptor antibody encephalitis but not at other ionotropic receptors. Moreover, though these changes are observed across brain regions, these effects predominate at the NMDA receptors of excitatory neurons rather than at inhibitory interneurons. Given that EEG is a ubiquitously available clinical method, our findings suggest a unique re-purposing of EEG data as an assay of brain network dysfunction at the molecular level.

## Teaser

Patients with anti-NMDA-receptor encephalitis display diverse early symptoms and no clear imaging marker of the disease is currently available. Symmonds *et al.* report that using EEG alone, it is possible to identify the common underlying abnormality in NMDA receptor function, facilitating early diagnosis and potentially improving patient outcomes.

See Roberts and Breakspear (doi:10.1093/brain/awy136) for a scientific commentary on this article.

## Introduction

*N*-Methyl-d-aspartate receptor (NMDAR) antibody encephalitis, first described in 2007, is an autoimmune disorder with diverse psychiatric and neurological features, most commonly psychosis, disorientation, amnesia, seizures and a complex movement disorder (Dalmau *et al.*, 2011). EEG is frequently performed in initial clinical investigations to assess the degree of encephalopathy and to detect the presence of seizures (Irani *et al.*, 2010*b*). EEG findings can vary from normal to encephalopathic without any specific diagnostic indicators although a ‘delta-brush’ (Schmitt *et al.*, 2012) pattern has been described in some patients. MRI findings too, are likely to be normal in the majority of patients (Irani *et al.*, 2010*b*). The diagnosis of NMDAR-antibody encephalitis is based on a typical clinical phenotype, with the demonstration of autoantibodies in CSF and serum. However, clinical presentations can be highly heterogeneous, making diagnosis challenging (Graus *et al.*, 2010; Jézéquel *et al.*, 2017). Improved short- and long-term clinical outcomes are dependent upon early institution of immunotherapies, which aim to reduce autoantibody levels and the accompanying immune-system activation (Gabilondo *et al.*, 2011). A non-invasive measure of NMDAR function could thus facilitate early diagnosis and treatment, and provide the ability to track therapeutic response with a specific biomarker of disease activity. Overall, the ability to non-invasively assay synaptic ion channel function in clinical settings would translate our growing molecular understandings of psychiatric and neurological diseases into novel tools for clinical practice (Lisman, 2012; Friston *et al.*, 2016*a*). If successful, such assays would allow for detecting ion channel disruptions across a wide class of illnesses, not limited to those with an immunological aetiology.

NMDAR-antibody encephalitis provides an ideal testbed for developing a specific non-invasive measure of ion channel dysfunction *in vivo* in humans. Here we present evidence for identifying aberrant channel signalling using dynamic causal models (DCMs) for EEG-derived cross-spectral recordings (Moran *et al.*, 2008). DCMs use neurobiologically-interpretable mathematical models of neuronal ensembles to predict fluctuations in synaptic currents and their influence on postsynaptic membrane potentials as measured with EEG. The model comprises three levels of description. At the lowest level, dynamic equations describe the flow of particular ions at the synapse. Then, at the population level, interactions of groups of synapses describe a connected local circuit. Finally the summed local circuit currents are adjusted using appropriate forward models to mimic transmission from the neuronal source through the scalp to the recording electrode (Kiebel *et al.*, 2006). Crucially, DCM allows for solving the ‘inverse problem’ of inferring, from individual patient recordings, the most likely biophysical parameters that generated the measured brain activity.

Here we tested whether this approach can reveal the specific ion channel abnormality in 29 patients with NMDAR-antibody encephalitis. We aimed to determine whether NMDA parameter estimates are selectively identified as abnormal, using standard clinical EEG and a DCM that incorporates signalling at NMDA, AMPA and GABA_A_ receptors. We also aimed to test whether particular cell types were more or less affected. In what follows we first show that cross-spectra generated from spontaneous (‘resting state’) EEG measurements from patients with NMDAR-antibody encephalitis exhibited differences when compared to encephalopathic patient controls (a range of causes of encephalopathy without NMDAR antibodies, *n = *18) and neurological patient controls (*n = *18) who underwent a clinical EEG. We then show that these spectral differences could be captured by DCM, with subject-specific model parameters reflecting the underlying mapping from ion channels to EEG spectra. Using an empirical Bayesian approach (Friston *et al.*, 2016*b*) we revealed that the effect of NMDAR-antibody encephalitis is expressed selectively in the DCMs’ inferred NMDAR parameters, in line with the underlying synaptic abnormality.

## Materials and methods

### Patient diagnosis and clinical status

Retrospective data collection had research approval from Oxford University Hospitals NHS Trust. Anonymized retrospective EEG were collected from patients with a clinical diagnosis of NMDAR-antibody encephalitis based on one or more typical clinical features (abnormal psychiatric behaviour or cognitive dysfunction, speech dysfunction (pressured speech, dysphasia/mutism), seizures, movement disorders (dyskinesia, rigidity, dystonias), decreased consciousness, autonomic dysfunction) and positive serology for NMDAR antibodies during their illness (either serum and or CSF antibodies to the GluN1 subunit of the NMDAR) (Graus *et al.*, 2016). They underwent routine EEG at the tertiary centre either early in the course of their illness or following assessment for relapse or persistent symptoms and positive serology despite immunosuppressive treatment. In addition, data were collected from patients with encephalopathy from a range of metabolic, inflammatory and structural causes, as well as a separate cohort of non-encephalopathic patients with neurological symptoms requiring an EEG as part of standard clinical management. Clinical details of included patients are summarized in Supplementary Tables 1–3.

### EEG acquisition and preprocessing

Routine clinical EEG data had been collected from adults (inpatients and outpatients) at rest for 30–60 min. Acquisition used a standard 10-20 montage set-up with 21 channel recording and clinical-grade amplifiers (Natus). Impedances of all electrodes were <5 kΩ prior to data acquisition. During acquisition, data were sampled at 250–500 Hz. Data were exported and epoched into 5-s segments and visually inspected to remove epochs containing large baseline shifts, eye blink or movement artefacts, or overt discharges using the Fieldtrip toolbox in MATLAB (Mathworks, Natick, USA). This resulted in an average of 12 min of wake resting state data post artefact correction (∼144 trials of 5-s epochs). These data were low-pass filtered at 42 Hz. For data processing we used the analysis routines available in the academic open source software SPM12 (Wellcome Trust Centre for Neuroimaging, London, UK, http://www.fil.ion.ucl.ac.uk/spm/). These 21-channel sensor data were decomposed into eight spatial principal modes to reduce complexity in model fitting using singular value decomposition.

### Spectral analysis

To summarize the epoched resting state data and obtain data features for model fitting, we used a sensor-space representation. We specifically retained eight singular vectors that summarized the spatial distribution of responses on the scalp. Using these time series we then applied an autoregressive method developed to calculate the complex cross spectral energy across these eight spatial modes, including the real auto-spectral energies and complex cross-spectral energies. This produced a symmetric 8 × 8 matrix of cross-spectral energy in sensor space. Thus, we had a matrix of 1856 real and 1856 complex data points to fit for each subject for the beta-gamma band DCM (20–48 Hz) and 320 real and 320 complex data points to fit for each subject for the delta-theta (2–6 Hz) band DCM. To produce the model-based recapitulation of these data we first generated data in source space with the model using the dynamics described in Supplementary Fig. 7. We then passed this 4-source data through the leadfield, calculated from a finite element model (SPM’s standard EEG leadfield procedure) and reproduced the spatial modes with an identical mixture of channels as the original data-driven solution. Thus, we fit the model based on these sensor-level data and sensor-based modelled replications. We illustrate this procedure in full in Supplementary Fig. 1.

We summarized model fit as per cent variance explained (i.e. R^2^). This was calculated as the squared correlation coefficient between the data-derived and the model-predicted complex cross-spectral responses (we concatenate the matrix of cross spectral responses into a vector of absolute values to perform the calculation).

To test for summary differences over groups in the beta-gamma spectra we used an analysis of variance over the average absolute power from 28 to 40 Hz. This power measure was taken from the first principal mode’s autospectral response. For the shorter delta-theta band we performed a summary analysis on the average response from 2 to 4 Hz, here taking the second principal mode. To investigate the direction of group effects *post hoc*, we applied *t*-tests and corrected for multiple comparisons.

### Dynamic causal modelling

In DCM the neural mass models have been developed based on known cortical anatomy and physiology. We implemented a conductance-based model, with brain regions modelled as interconnected neural masses with specific inter- and intra-regional connectivity. These same models have been previously extensively applied to electrophysiological data in animals and humans (a full discussion and overview of the development and implementation of these models can be found in Marreiros *et al.*, 2009; Moran *et al.*, 2013).

We used the canonical microcircuit model (CMC) for DCM (Bastos *et al.*, 2012), with NMDARs (spm_fx_cmm_nmda), which accounts for laminar differences in the origin of forward and backward connections in the brain (superficial versus deep pyramidal cells, respectively). We applied anatomical priors corresponding to a default mode (resting state) network with four source locations based on previous MEG reports (Baker *et al.*, 2014), with left and right parietal cortical sources at MNI locations [−29 −68 49] and [29 −68 49], respectively, and left and right prefrontal sources at MNI locations [−33 45 28] and [33 45 28], respectively (Fig. 2A). The CMC that we used comprised excitatory and inhibitory extrinsic connection parameters from four distinct cell layers: superficial pyramidal cells, spiny stellate cells, deep pyramidal cells, and inhibitory interneurons (Fig. 2B). Within the model, superficial pyramidal cells in the parietal sources carry signals to spiny stellate cells and deep pyramidal cells in a feedforward manner up the cortical hierarchy, while deep pyramidal cells carry top-down signals in a feedback manner from frontal cortex to both superficial pyramidal cells and inhibitory interneurons in the parietal sources.

In terms of the physiological mechanisms of connectivity, the dynamics of postsynaptic responses are modelled as capacitive synaptic current flow across the synaptic membrane; a summary of generic equations dictating the dynamics of one cellular population are shown in Supplementary Fig. 7, and detailed equations can be found in Moran *et al.* (2011*b*). The active currents across the postsynaptic membrane include ligand-gated inhibitory (Cl^−^) and excitatory (Na^+^/Ca^2+^) and ion flow mediated by fast GABA_A_ and AMPA receptors and slower NMDARs, respectively. Each neuronal subpopulation has one voltage/membrane-depolarization state and three conductances (AMPA, GABA_A_ and NMDA). Thus, each subpopulation has four states. With four populations per source and four sources, which amounts to 64 coupled ordinary differential equations (ODEs) for a given inversion. Hence there are 64 potential ‘poles’—regions of infinite frequency response that are solutions to the characteristic equation of the dynamic system—which are all mixtures of the model parameters. The magnesium block at NMDARs is voltage-gated and non-linear. In our model the NMDAR conductance is thus augmented by a parameterized non-linear sigmoid gain function (Supplementary Fig. 7, with three parameters as proposed by Durstewitz *et al.*, 2000). Reversal potentials for sodium, calcium and chloride were fixed at 60 mV, 10 mV and −70 mV, respectively. A potassium leak current was used to account for all passive ionic currents, with V_L (reversal potential) −70 mV. The model also included a driving current input, which enters the spiny stellate cells. The conductances of these ligand-gated ion channels are dynamic states in our model described by differential equations (Supplementary Fig. 7). The conductance reflects the number of open channels, which depends upon the coupling of presynaptic input to the postsynaptic response, and the channel’s time constant with prior values of 12 ms, 8 ms and 100 ms, for AMPA, GABA_A_ and NMDARs, respectively. Importantly, the inverse of the time constants are known as rate constants and are critical parameters of our model as they represent the rate of channel opening. The presynaptic firing is controlled by the membrane potential of the efferent cell population. The specific code for this implementation can be found in the academic freeware in http://www.fil.ion.ucl.ac.uk/spm/toolbox/DCMMEEG.

For the DCM analyses, EEG activity were fitted over two frequency bands (20–48 Hz and 2–6 Hz) using two identical DCMs. DCM optimizes a posterior density over free parameters (parameterized by its mean and covariance) via a variational Bayesian inversion procedure giving a trade off in accuracy and complexity (Friston *et al.*, 2007). To set prior parameter values we inverted DCMs for three neurological patient controls selected at random and used their average posterior parameter values as prior values for DCM inversions of all other subjects in all groups. This was to ensure we had found a (local) maximum, i.e. an optimum parameter setting for the groups generally and prevented any bias in model parameters between the groups. We harvested the parameter estimates from optimized DCMs for the two models and used a canonical variates analysis (spm_CVA) to assess group differences amongst parameter sets. These sets comprised parameters specific to NMDA, AMPA and GABA_A_ receptors (Supplementary Fig. 3 and Supplementary Table 4). Canonical variates analysis is a general method subsuming (multivariate) linear regression, canonical correlation analysis, multivariate analysis of variance, discriminant analysis, and Hotelling’s T-test (Hair *et al.*, 1998). Subject-specific estimates of the model parameters across groups are submitted to a linear analysis testing specifically for group differences. The results of this produce a principal canonical vector (weights over parameters) and canonical variate (weights over subjects). The canonical vector specifies the weights of the parameter mixture that yields the greatest correlation with the mixture of explanatory variables (here, group differences). That is, the canonical vector provides the optimal contrast over parameters to yield the maximal group differences.

For the Bayesian analysis, we applied a recent second level modelling extension to DCM that allows for random effects across groups. This so called parametric empirical Bayesian scheme refits a ‘full model’ (where all parameters can co-vary according to group designation) and produces reduced models where all smaller combinations of parameter variation are considered and informed by the group averages. Here we apply two group designations, presence or absence of NMDAR-antibody encephalitis diagnostic label and presence or absence of encephalopathy. These two groupings were included in our second-level design matrix, which had three columns. The first column represented the average effect (and contained ones), the second column described the encephalitis effects (and comprised ones for NMDAR-antibody encephalitis patients, ones for other encephalopathy patients and zeros for non-encephalopathic patients), the third column described the effect of NMDAR-antibody encephalitis (and comprised ones for NMDAR-antibody encephalitis patients, zeros for other encephalopathy patients and zeros for non-encephalopathic patients). We were most interested in how the third column of the design matrix was represented by differences in the DCM parameters within our cohort. We hypothesized that the second effect (encephalopathy) should be represented by changes in multiple ion channels. We hypothesized that the third effect (of NMDAR-antibody encephalitis) should be represented by changes in NMDA parameters only. We report those parameters that exhibit significant effects of NMDAR-antibody encephalitis and those parameters that exhibit significant effects of encephalopathy using a probability of *P* > 0.95. Of note, here we examine parameter-specific rather than model-specific effects. The underlying free energy of the parametric empiric Bayes model pertains to all the subject data from all the groups, assuming random parametric effects at the between-subject level as well as group-specific effects in the hierarchical model (Friston *et al.*, 2016*b*).

## Results

### Resting state power spectra exhibit group differences

**Figure 1:**
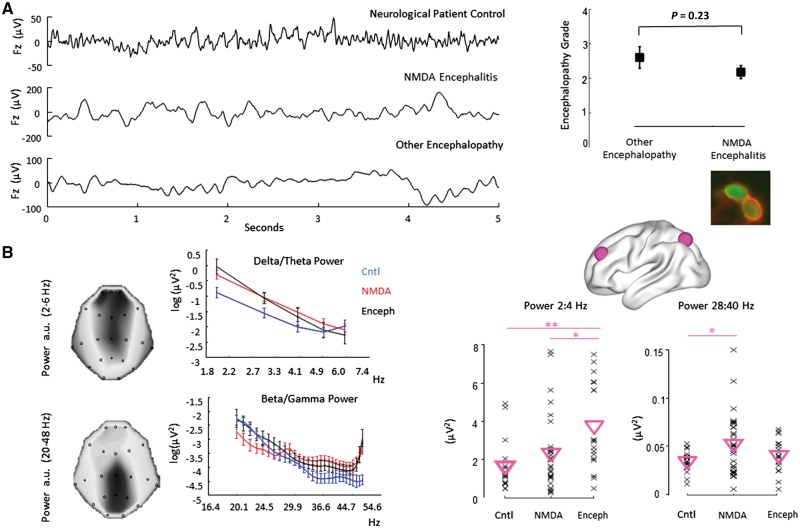
**Spectral characteristics of NMDAR-antibody encephalitis.** (**A**) *Left*: EEG, scalp data from mid-frontal channel Fz from exemplar patients in each group. *Right*: Though NMDAR-antibody encephalitis can present with a range of clinical and electrographic features there was no statistical difference in the clinical severity of encephalopathy (West-Haven scale) applied to the two groups. *Inset* shows merged fluorescence image of cell-based assay of positive NMDAR antibodies in serum. (**B**) On the *far left*, scalp maps indicate the 21 sensor locations and power estimates from two exemplar subjects. In the *left* panel we show the spontaneous delta-theta and high beta-gamma power averaged across the groups. Given previous reports of altered low (delta/theta) and high (beta/agama) frequency power in patients with NMDAR-antibody encephalitis, we tested these ranges and found a significant effect of group [*right* panel (log-log plot of spectral power)]. For delta responses the spectral differences were driven by enhanced power from 2 to 4 Hz in the control encephalopathic patients (ANOVA: F = 6.0, *P = *0.004). For the beta/gamma band it was the NMDAR-antibody encephalitis group that exhibited the greatest power (ANOVA: F = 3.63; *P = *0.03). The average response over the band is plotted for each subject (x’s) with inverted triangles indicating the group mean.

The data in the current study comprised resting state, eyes open, recordings acquired in one sitting, from 29 patients with NMDAR-antibody encephalitis (Supplementary Table 1), 18 encephalopathic controls (with encephalopathy non-related to a specific autoimmune channelopathy, Supplementary Table 2) and 18 neurological patient control participants (Supplementary Table 3). We investigated the cross-spectrum of these recordings, which summarizes longer, spontaneous recordings (Fig. 1A) into a set of second-order auto and cross-correlation statistics (Fig. 1B), where fits are performed on sensor-space, complex, cross spectral energies (Supplementary Fig. 1). We aimed to determine whether particular frequencies could be discriminated across the groups for subsequent DCM analysis, including high frequency beta-gamma band responses (20–48 Hz) and lower frequency delta-theta band responses (2–6 Hz), as in previous EEG findings on NMDAR-antibody encephalitis (Dalmau *et al.*, 2011; Schmitt *et al.*, 2012).

The patients with NMDAR-antibody encephalitis had behavioural changes ranging from subtle encephalopathic features to gross obtundation or unresponsiveness, which were graded using the West Haven encephalopathy scale (Supplementary Table 5). Overall, there was no difference in clinical severity of encephalopathy between the NMDAR-antibody encephalitis compared to the encephalopathy group (Fig. 1A). However, there were differences in the cross spectrum at particular frequencies. In the delta-theta band, an analysis of variance revealed significant differences in power at low (2–4 Hz) frequencies (F = 6.0, *P = *0.004; Fig. 1B), with a *post hoc* analysis showing significant increases for the ‘other’ encephalopathy group compared to the neurological patient controls (*P = *0.003 corrected) and compared to the NMDAR-antibody encephalitis group (*P = *0.043 corrected). At higher frequencies (28–40 Hz) we also observed significant differences between the three groups (F = 3.63; *P = *0.03) with increased beta-gamma power present in the spontaneous spectrum of patients with NMDAR-antibody encephalitis relative to neurological patient controls (*P* = 0.03 corrected, Fig. 1B).

**Figure 2:**
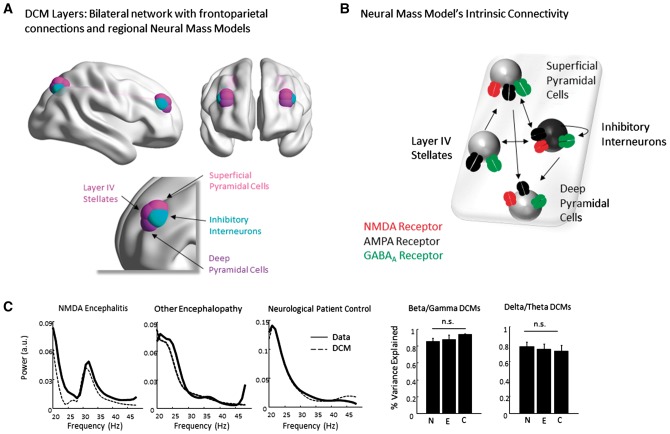
**The DCM.** (**A**) Sources for the DCM are based on MEG ‘default mode’ studies and comprise two parietal and two frontal sources. These are connected with forward connections (from supragranular layers) from parietal to frontal cortex and with backward connections (from infragranular layers) from frontal to parietal cortex. (**B**) Each source is populated with a four-population neural mass model comprising supragranular pyramidal cells, inhibitory interneurons, layer IV stellate cells and infragranular pyramidal cell layer. Receptors and intrinsic connections represented in the neural mass models are shown. Our dynamics prescribe changes in postsynaptic membrane potential based on the dynamics of ion channel transmission (Supplementary Fig. 4). These ions are controlled by conductances representing binding to GABA_A_, NMDA and AMPA receptors. Each of these receptors is imbued with a physiologically plausible time constant, which acts as the inverse of a rate constant controlling the rate of opening and closing. Each channel also has its own reversal potential set at physiological levels (see text). (**C**) The DCMs produce spectra, which recapitulate the patterns of beta-gamma and delta-theta responses observed in the empirical recordings. Importantly, no group effects of fit were observed. In other words, the model was equally applicable to all data. C = patient controls; E = other encephalopathy; N = NMDA encephalitis.

These sensor-level group differences in EEG activity established an explanandum to which we could apply our DCM with ion channel parameters. Using data from the delta-theta band and data from the beta-gamma band, we sought to fit two DCMs (Fig. 2) to each set of subject-specific spectral responses, creating a multivariate mapping from model parameters representing dynamics at NMDA, AMPA and GABA_A_ receptors to these spectral data features.

### Model fits capture spectral responses

Given our resting state paradigm, we used *a priori* source locations based on a previous comprehensive study of resting state magnetoencephalographic networks (Baker *et al.*, 2014), which showed prominent bilateral parietal and medial frontal gyrus activity at rest—commensurate with the default mode network observed in functional MRI studies (but without a precuneus source) (Brookes *et al.*, 2011). Thus four connected sources, with four interhemispheric connections, were used to describe delta-theta and beta-gamma cross-spectrum observed at the scalp sensors (Fig. 2A): with parietal sources sending forward connections to frontal sources and frontal sources sending backward connections to parietal sources within each hemisphere (Moran *et al.*, 2008). Each source comprised a neural mass model that had four neuronal subpopulations (superficial and deep pyramidal cells, inhibitory interneurons and layer IV stellate cells) with dynamics determined by intrinsic connections mediated via NMDA, AMPA and GABA_A_ receptors (Fig. 2B). These models have been extensively developed and used for modelling electrophysiological data in humans and animals (for a review see Moran *et al.*, 2013). Of particular note, NMDARs have relatively slow time constants compared to AMPA receptors (12 ms versus 100 ms), which leads to distinct dynamical properties. Model inversion proceeds by optimizing the model free energy, a bound on the model evidence, using a variational Bayesian estimation scheme, which accounts for dependencies amongst parameters (Supplementary material). Together, dynamics along these intrinsic (between subpopulations within a source) and extrinsic (between sources) connections could reproduce the scalp-based EEG recordings (Fig. 2C). We found that our beta-gamma models provided excellent fits to the data with an average per cent variance explained of 86%, 88% and 94% for our NMDAR-antibody encephalitis, other encephalopathy and neurological patient controls, respectively, and no significant differences in fit across the groups (Fig. 2C). For the delta-theta models the variance explained was lower with 77%, 74% and 71% variance explained for the three groups.

We also performed a comparison with a model including an interconnection from the superficial pyramidal cells to the layer IV spiny stellate cells, given that stellate cells also express NMDARs (Moran *et al.*, 2013). We fitted this new model, with the additional connections to all of our dataset, and then performed a statistical model comparison (Supplementary Fig. 2). Interestingly, this revealed that our original model was favoured when fitting spectral responses in the beta and gamma range (consistent with the literature hypothesizing a pyramidal-interneuron gamma mechanism for high frequency oscillations) (Wulff *et al.*, 2009; Gonzalez-Burgos and Lewis, 2012), while the augmented models with NMDAR input to layer IV cells was favoured when fitting the delta-band spectrum. This demonstrates that the estimates are robust to changes in model structure, but provides additional insight that accounting for NMDARs on stellate cells improves the ability of the model to explain slow frequency responses in our patients. Importantly, however, this effect does not improve discrimination between NMDAR antibody encephalitis and encephalopathic control patients in our sample. Crucially there were no significant group differences in data fits in the delta-theta models (Fig. 2C) either. Thus, we show that the models comprising four sources (each with four neuronal subpopulations) recapitulated the original EEG data.

### Ion channel measures: specific group differences in NMDA parameters

We assessed whether the optimized model parameters reflected the true underlying synaptic deficit. In particular, we sought to determine whether decreased receptor signalling at NMDARs in patients with NMDAR-antibody encephalitis would be represented by a specific abnormality in parameters that control NMDAR conductances in the model. Each DCM comprised 41 neuronal parameters. These included: a rate constant for each of the three receptors (NMDA, AMPA and GABA_A_) in each of the four sources (3 × 4 parameters); an intrinsic connection strength describing the weight of presynaptic firing on each of the three postsynaptic receptors (3 × 4 parameters), with separate weights for NMDAR-mediated inputs to inhibitory and excitatory neurons (four parameters); an extrinsic connection strength describing the weight of forward connections, i.e. presynaptic parietal firing on postsynaptic frontal responses at AMPA receptors for both hemispheres (two parameters), and similarly for backward connections (two parameters); a parameter describing the variance in ensemble firing (one parameter); a parameter describing background current (one parameter); three parameters controlling the magnesium block, voltage-dependent non-linearity of the NMDA channels (three parameters); and finally, a time delay for synaptic transmission within each of the four sources (four parameters). Alterations in these model parameters have non-linear interactive effects on model dynamics. In Supplementary Fig. 7 we simulate the individual and dual effects of non-linearity and slower time constants of the modelled NMDAR. Interestingly both dynamic components of the channel alter the spectra output of the neural mass revealing a non-isomorphic relationship between spectral frequency content and time constants of the receptor.

**Figure 3:**
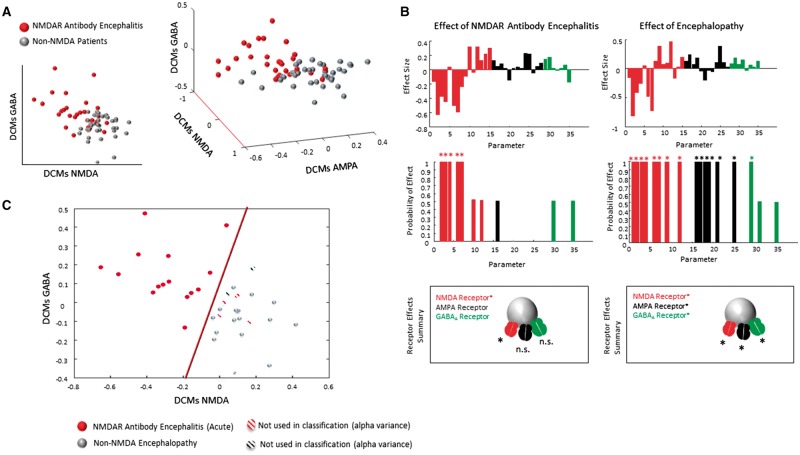
**Receptor fingerprints from DCM parameters.** (**A**) Our DCMs contained parameters relating to three major receptor types, namely NMDA, AMPA and GABA_A_. Here we show the linear combination of each parameter set that best described the partition amongst the groups. Our plots show each parameter scaled by the canonical vector (Supplementary Fig. 1). Each of our patients are represented by either a red sphere (NMDAR-antibody encephalitis diagnosis) or a grey sphere (for both other encephalopathy and neurological patient controls). Statistically, only the NMDA parameter sets showed any significant multivariate difference between groups. (**B**) Using parametric empirical Bayes, we tested for a group difference of NMDAR-antibody encephalitis while accounting for the presence or absence of encephalopathy in general using all patient records. We used a model search across group-effects to determine which parameters in our DCM showed significant group differences both in terms of NMDAR-antibody encephalitis status (present in 29 records) and encephalopathy status (present in 47 records). The *top* bar charts show the Bayesian covariate, which are reduced via model comparison to leave only those significant effects for each group class. The lower bar charts indicate group effect probabilities for each parameter with a significance limit set to >0.95 probability. The *insets* highlight the set of receptors that exhibit significant group effects. We show that only the NMDA parameters show significant effects for the NMDAR-antibody encephalitis while all three ion channels are significant predictors of encephalopathy. (**B**) To investigate individual patient classification, we tested NMDAR-antibody encephalitis patients using only those EEG records obtained within the ‘acute phase’ (<3 months since symptom onset, *n* = 19, see Supplementary Table 1). Fifteen of these 19 patients (red spheres) could be distinguished from the encephalopathic controls (grey spheres). We determined that the misclassifications (striped symbols) could be attributed to a high amplitude occipital alpha rhythm in occipital regions (Supplementary Fig. 5). By accounting for this confound, an accurate class label could be applied to all of our acute patients.

Thus, for each model, we could order our parameters into three ion-channel sets, a set of NMDAR parameters (15 parameters in total), a set of AMPA receptor parameters (12 parameters in total) and a set of GABA_A_ receptor parameters (eight parameters in total, Supplementary Table 4). To determine whether particular ion channel parameters exhibited significant group differences, we first used a canonical variates analysis (CVA) on the *a posteriori* expectations (model estimates) of the parameters (Fig. 3 and Supplementary Fig. 4). Specifically, we were interested in a significant mapping from the multivariate parameter estimates from each ion channel set to the diagnosis of NMDAR-antibody encephalitis. Three CVAs for each set of NMDA, AMPA and GABA_A_ were thus conducted to test for significant differences between patients with or without NMDAR-antibody encephalitis. The parameter sets for each ion channel comprised optimized individual estimates from both the delta-theta and beta-gamma bands. We found that for the set of NMDAR parameters, the group means, between those with and without the diagnosis of NMDAR-antibody encephalitis were significantly different (*P = *0.047; χ^2^ = 25.17). Importantly, when these tests were applied to the AMPA receptor parameter sets, no group differences in our multivariate response variables were observed (*P = *0.29; χ^2^ = 17.4). Similarly, the analysis reported no group differences in GABA_A_ receptor parameter values (*P = *0.56; χ^2^ = 13.4). Thus we identified a group effect of NMDAR-antibody encephalitis that was selective to the NMDAR parameter set using DCMs that map from neuronal synaptic dynamics to empirical EEG recordings. In Fig. 3A we display the individual parameters from each set (NMDA, AMPA and GABA_A_) for each participant—where the aggregate NMDA parameter effect is shown by weighting each individual’s estimate set with the canonical vector for the corresponding channel. The group separation is predominantly in terms of NMDA parameters (Fig. 3A and Supplementary Fig. 3). Here we also observe that the connectivity parameters decrease, commensurate with the hypothesized pathophysiological receptor hypofunction in this autoimmune disorder.

To serve as a useful clinical tool, these aberrant synaptic parameters should be distinguishable on an individual level. For our individual-level analysis we selected a subset of our NMDAR-antibody encephalitis patients. Specifically, we selected only those patients whose EEG was acquired during the initial acute phase of the illness (<3 months since symptom onset, Supplementary Table 1). This set included 19 of our total 29 patients, with the 10 removed patients corresponding to chronic or recovering phases of the illness (Supplementary Table 1). When compared to non-channel related encephalopathy, 15 of these 19 patients showed a linear separation based on the DCM parameters (Fig. 3B). Importantly, we were able to identify, *post hoc*, the mischaracterization of those four patients as having prominent high occipital alpha power (Supplementary Fig. 5). If we account for alpha power on the scalp, we can successfully predict the clinical diagnosis of our entire patient cohort in the acute phase of illness.

### Group effects: a parametric empirical Bayesian approach

To conclude that AMPA and GABA_A_ receptors are not affected by the presence of NMDAR-antibodies, a Bayesian analysis is required that allows us to ‘accept the null’ hypothesis. In other words, a Bayesian analysis allows us to establish the absence of a group effect. Thus, we applied to these models an analysis that returns the probability of an effect at all parameters (rather than the probability of no-effect as in the classical statistical CVA-based test above). This Bayesian approach uses parametric empirical Bayes (Friston *et al.*, 2016*b*), which allows for random effects of model parameters at the group level. In order to compute these probabilities, the parametric empirical Bayes scheme performs a search over all possible group (second-level) effects—postulating that any combination of parameters might deliver an effect of NMDAR-antibody encephalitis. This approach effectively re-estimates model parameters at an individual level for any combination of group effects and uses Occam’s razor to reduce the second level effects until only meaningful parameters that contribute to group differences are retained (Fig. 3C). We tested for the most parsimonious model that exhibited group effects both in terms of an effect of NMDAR-antibody encephalitis and in terms of the presence of encephalopathy. Remarkably, for the beta-gamma models the significant group effects of NMDAR-antibody encephalitis were observed solely in NMDA parameters. While in contrast, the group effect of encephalopathy was expressed across our range of receptors–as would be expected from our heterogeneous encephalopathy aetiologies (Fig. 3C and Supplementary Table 2). In particular five NMDA parameters showed significantly decreased function in the anti-NMDAR encephalitis group. These parameters were the rate of channel opening and closing at the right parietal, right prefrontal and left prefrontal sources as well as the intrinsic connection to NMDARs at excitatory neurons in the right parietal and left prefrontal source. For the delta-theta models no significant group effects were observed (Supplementary Fig. 6), indicating a lack of discriminability across NMDA condition for this data feature.

### Effects on inhibitory interneurons

One prominent hypothesis is that direct NMDAR hypofunction in inhibitory interneurons leads to disinhibition in corticolimbic regions, underpinning symptoms in schizophrenia (Olney *et al.*, 1999; Benes and Berretta, 2001; Lewis *et al.*, 2004; Woo and Crowell, 2005; Coyle, 2006; Homayoun and Moghaddam, 2007; Nakazawa *et al.*, 2012). However, findings remain mixed in schizophrenia (Schwarcz *et al.*, 2001; Volk *et al.*, 2002), and *in vitro* studies of the effect of patient-derived NMDAR antibodies (Hughes *et al.*, 2010; Moscato *et al.*, 2014) have shown broad effects on both excitatory and inhibitory neurons, with no cell type selectivity. The alternative hypothesis is that NMDAR hypofunction on pyramidal cells consequently leads to disrupted efferent drive to interneurons and causes abnormal synchronization of feedback inhibition and firing in the inhibitory interneuron population (Gonzalez-Burgos and Lewis, 2012), in turn leading to pyramidal cell hyperexcitability. This accords with the relatively weak NMDA contribution to fast synaptic activation of inhibitory interneurons (Nyiri *et al.*, 2003; Rotaru *et al.*, 2012), and animal models of cell-specific NMDAR effects (Billingslea *et al.*, 2014).

To investigate this mechanism further in our model, we examined the predicted firing pattern of the inhibitory cell populations by simulating their population activity and the spectral output (Supplementary Fig. 9). It is important to note that the spectral output of the inhibitory interneurons does not directly contribute to scalp-measured EEG because of their dendritic arborization topology. However, we can readily simulate their contributing dynamic at the level of the neural mass directly. Interestingly, this shows that in our patients with NMDAR-antibody encephalitis, there is a suppression of inhibitory cell firing across the beta band compared to normal controls. These cell-specific spectral changes provide support for the hypothesis that there is decreased inhibitory cell synchronization as a knock-on effect of pyramidal cell NMDAR hypofunction. Unlike the consistent effect seen on the pyramidal cell NMDA conductance, there is no consistent parameterization of this spectral effect in the inhibitory cells (i.e. it arises from a mixture of non-linear parameter effects). Nonetheless we can recover this effect through simulating the dynamics of this cell population.

### Effects on system stability

We also considered whether the present pathological combination of parameters leads to a more unstable system in the NMDAR antibody encephalitis patients. The eigenspectrum from the Jacobian shows, as anticipated, that the NMDAR parameterizations result in closer to unstable dynamics compared to the other groups (Supplementary Fig. 8).

## Discussion

Our results suggest that standard clinical EEG can serve to accurately reveal unobservable synaptic pathology, utilizing a biophysical model of connected cortical ensembles and Bayesian inversion techniques. We first identified a specific change in the spectral characteristics of resting-state EEG signals in patients with NMDAR-antibody encephalitis. We then used these spectral signatures to constrain individual models of ionotropic receptor function (Friston *et al.*, 2012). The parameter estimates, recovered from DCMs of the cross-spectral densities, were used to identify the functional status of NMDA, AMPA and GABA_A_ receptors. This revealed, using a Bayesian approach, a specific effect in NMDAR conductance in a patient group in which NMDA antibodies had been detected and in which NMDAR-antibody encephalitis had been the final diagnosis. Crucially, we were able to identify, based on individual parameter estimates, patients in the acute phase of the illness with high sensitivity when accounting for effects in the alpha band. We could discriminate the NMDAR-antibody encephalitis patients from patients with a diverse range of encephalopathic illnesses. These findings provide novel evidence for the diagnostic and prognostic capabilities of clinical EEG using a model-based approach, and extend our previous findings on magnetoencephalographic-based channelopathy detection in genetic channelopathy case studies (Gilbert *et al.*, 2016).

Our analysis is motivated by previous neuroimaging studies of patients with NMDAR-antibody encephalitis that have revealed deficits in resting state functional MRI connectivity in the default mode network (Finke *et al.*, 2013) and by studies in the electrophysiological domain that have revealed distinct patterns in spectro-temporal EEG characteristics. Specifically, a feature reminiscent of the neonatal ‘delta-brush’ of prematurity has been identified that is associated with prolonged hospitalization (Schmitt *et al.*, 2012) and potential for status epilepticus (Herlopian *et al.*, 2016). This feature comprises enhanced 20–30 Hz (beta band) signals superimposed upon a delta rhythm (Van Noord *et al.*, 2016). Here we analysed both of these high and low frequency bands within nodes of the default mode network (Brookes *et al.*, 2011) and characterized the neurophysiological processes that could generate the observed response in these bands (Moran *et al.*, 2008). Interestingly, our model-based estimates of NMDAR function in patients with NMDAR-antibody encephalitis produce less stable dynamics, which may reflect the increased propensity to seizures in these individuals. This alteration in low and high frequency bands and their coupling may contribute directly to the early cognitive and perceptual changes observed in NMDAR-antibody encephalitis. In particular, psychosis is a common symptom that occurs early in the disease (Kayser and Dalmau, 2016). We have previously used DCM in a rodent ketamine model of psychosis (Moran *et al.*, 2015) to show that enhanced high-frequency oscillations are associated with NMDAR-based reduction in brain connectivity. This loss of NMDAR function may thus, overall, contribute to a loss in signal integration among brain regions, which our results suggest may be mediated by a combination of effects on pyramidal cells and a consequent suppression of inhibitory interneuron activity. Moreover, this observation of enhanced high-frequency neuronal activity may relate to the hypermetabolic processes observed in PET studies of these patients (Leypoldt *et al.*, 2012; Heine *et al.*, 2015). In our statistical analyses (Fig. 3), we characterized the overall network response of these receptors. We discriminate effects of NMDAR dynamics (parameters specifying NMDAR time constants and non-linear voltage gating) from effects on NMDAR conductance (modelled by cell- and region-specific parameters). However, the dynamic causal model is agnostic as to the exact mechanism of NMDAR hypofunction. We localize effects to a decreased NMDAR conductance, but cannot discriminate between functional antagonism of receptors versus receptor internalization, although the latter mechanism has significant experimental support (Moscato *et al.*, 2014). Future work will expand on the anatomical foci revealed by DCM parameters, which may map to the heterogeneities in clinical presentation of these patients.

Currently there is no available technology to assess the synaptic components of cortical neuronal networks in humans non-invasively. Whilst limited assessments of neurotransmitter and receptor levels can be made using magnetic resonance spectroscopy and PET, respectively, these techniques do not directly measure synaptic activity and can be applied only to a limited set of neurotransmitters and receptors. NMDARs, which are crucial for plasticity and memory (Paoletti *et al.*, 2013), are among those ionotropic receptors for which quantitative investigation *in vivo* remain absent. Several types of ion channels delicately balance the excitation and inhibition of neurons to promote a global dynamic state at the edge of criticality (Deco *et al.*, 2013) and changes in excitatory/inhibitory ratios have been linked to neuropsychiatric diseases as diverse as autism (Yizhar *et al.*, 2011) and schizophrenia (Lisman, 2012). Aberrant function of a single ion channel can lead to various pathophysiological processes, where in the case of NMDARs, hyperactivity increases intracellular calcium, which can lead to excitotoxic cascades in ischaemic stroke (Shi *et al.*, 2013) and Alzheimer’s disease (Talantova *et al.*, 2013), while hypoactive signalling at inhibitory interneurons in the prefrontal cortex may contribute to abnormal circuit function in schizophrenia (Friston *et al.*, 2016*a*). Here we used one class of disease from a recently discovered family of autoimmune neurological disorders to provide a rare but remarkably specific ‘template disorder’ and lesion model, against which our non-invasive ion channel assay could be tested.

Autoimmune encephalopathies result from the endogenous production of antibodies against specific ion channel components, with a diverse range of antibodies against components of the NMDAR (Titulaer *et al.*, 2013), AMPA receptor (Graus *et al.*, 2010), LGI1 or CASPR2 (Tan *et al.*, 2008; Irani *et al.*, 2010*a*), glycine (Carvajal-González *et al.*, 2014) and GABA_A/B_ receptor (Pettingill *et al.*, 2015) all recently identified in patients with neurological and psychiatric symptoms. Current diagnostic assays for autoimmune encephalopathies involve serum and/or CSF analysis for antibody detection. In future extensions we aim to show that our model-based assay can distinguish receptor abnormalities between these autoimmune subtypes. Future developments of our modelling approach could also include structural connectivity (Heine *et al.*, 2015) measures from patients where we embed neural masses in anatomically-derived connected networks (Jirsa *et al.*, 2010).

These large-scale network models of brain activity contrast with our four-source DCM, where we trade-off network complexity for parameter identifiability of specific receptor types. The key value of the DCM method outlined here is related to the inversion of these models. We make use of these models in the forward direction—to generate data—but crucially, we also solve the inverse problem and identify parameter values on a patient-specific basis (Gilbert *et al.*, 2016). Our inversion routine reveals parameter covariances by stipulating a large multivariate Gaussian parameter space. We have previously shown that DCMs’ parameters co-vary within ranges that render them identifiable (Moran *et al.*, 2011*a*). This is reiterated here—since we demonstrate selective identification of an abnormal ion channel (at NMDARs), without significant effects present in AMPA or GABA_A_ receptor parameter values. In NMDAR-antibody encephalitis, cell cultures treated with patient CSF reveal a reduction in surface NMDAR density through receptor internalization (Hughes *et al.*, 2010), leading to a selective decrease in NMDAR-mediated currents without impairment of other synaptic processes (Irani and Vincent, 2011). It is these molecular mechanisms that are the most likely putative explanation for our model parameter changes. Although NMDAR activity can be modulated in a range of encephalopathic illnesses, clearly the deficits in NMDAR conductance in NMDAR-antibody encephalitis are severe and significant enough to yield an accurate biomarker allowing segregation of these patients from other matched encephalopathic control patients in our cohort.

Our current results from routinely acquired EEG data already demonstrate that our approach provides a feasible assay for individual clinical assessment. Given that EEG is ubiquitously available and cost-efficient, our results imply that neuro-modelling of EEG data could provide clinically useful tests for a wide range of brain disorders. Looking forward, our development of computational EEG fingerprints of individual NMDAR status may also yield new mechanistic insights into the aetiology and progression of other neurological and psychiatric diseases.

## Funding

M.S. is supported by a grant from the Academy of Medical Sciences. K.J.F. is supported by a Principal Research Fellowship from the Wellcome Trust. K.E.S. is supported by the University of Zurich and the René and Susanne Braginsky Foundation.

## Supplementary material

Supplementary material is available at *Brain* online.

## Supplementary Material

Supplementary Figures and Tables

## Abbreviations

DCM: dynamic causal model
NMDAR: *N*-methyl-d-aspartate receptor
