# Supplementary material for: Ion channels in EEG: isolating channel dysfunction in NMDA receptor antibody encephalitis
Source: Brain. 2018 Apr 30;141(6):1691–702. doi: 10.1093/brain/awy107 (PMC6207885; doi:10.1093/brain/awy107)
Supplement: Supplementary Figures and Tables [file awy107_brain-2017-01876-file005.pdf]

Supplementary Materials

List of Supplementary Materials

Supplementary Figure 1: Cross Spectral Energies – full data for fitting, sensor space and relation to model in source space

Supplementary Figure 2: Model comparison; Neural Mass Model with and without NMDA receptors on spiny stellate cells

Supplementary Figure 3: Canonical variate analysis

Supplementary Figure 4: DCM Parameters: Priors & Posteriors

Supplementary Figure 5: Alpha power in patients with encephalopathy.

Supplementary Figure 6: Delta/Theta model fits

Supplementary Figure 7: Conductance based neural mass model & NMDA Receptor effects

Supplementary Figure 8: Stability Analysis

Supplementary Figure 9: Spectral response at inhibitory interneurons

Supplementary Table 1: Clinical summary – NMDAR-antibody encephalitis patients

Supplementary Table 2: Clinical summary – non-NMDA encephalopathy patients

Supplementary Table 3: Clinical summary – non-encephalopathic patients

Supplementary Table 4: Parameter list for biophysical models with posterior parameter ranges.

Supplementary Table 5: Grading scales for electrographic and clinical features of encephalopathy

Supplementary Methods: Variational Bayesian procedure for fitting model parameters

Supplementary Figure 1

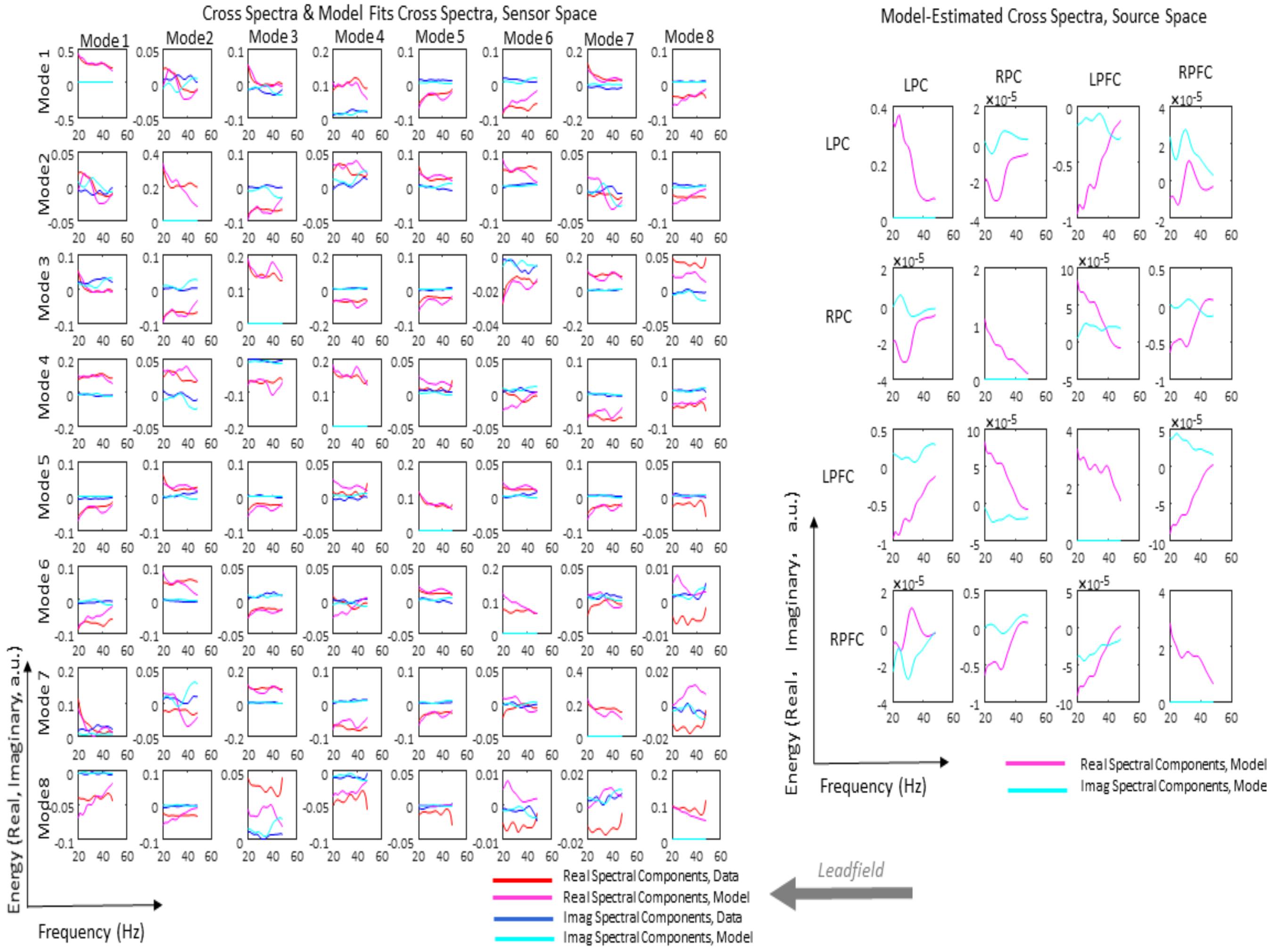

Supplementary Figure 1: Cross Spectral Energies – full data for fitting, sensor space and relation to model in source space

The model parameters were optimised based upon the error between data and model fit (with additional terms to account for model complexity – all contained within a single cost function known as the model's Free Energy). The actual data employed comprised the 8 principal spatial models of the sensor space time series using a singular value decomposition (SVD). Having obtained the 8 most independent channel mixtures we then calculated the complex cross spectral energy (left panel). The model in turn operates in source space (right panel) and also produces a spectral energy measure for each of the 4 sources – defined in MNI space. To obtain the

modelled data – i.e. that which is fit to the real data; we pass the source-space (right panel) to the modes in sensor space (left panel) via a leadfield and reconfigure the mixtures of the SVD. Thus model/data error is computed on the sensor-space data; a reduced/denoised SVD representation of the individual channels.

Supplementary Figure 2

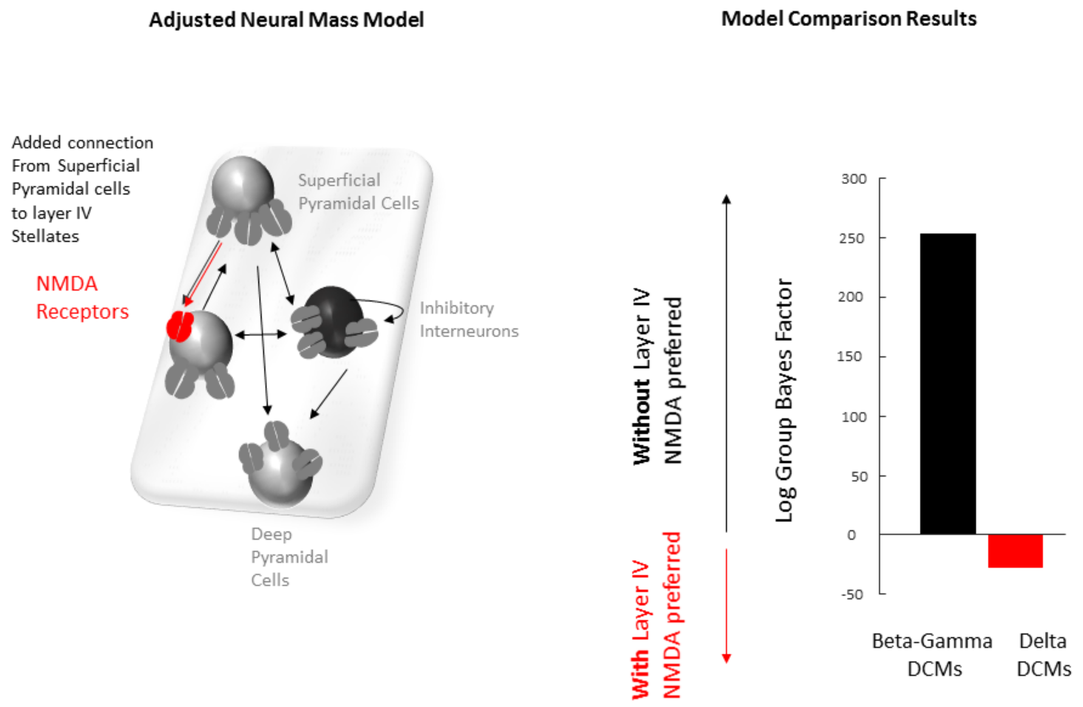

Supplementary Figure 2: Model comparison; Neural Mass Model with and without NMDA receptors on spiny stellate cells

In order to determine whether NMDA receptors at spiny stellate cells were important dynamics to include in our model, we augmented the neural mass to include NMDA receptor dynamics on this subpopulation also - and performed a Bayesian Model Comparison. By comparing the log group Bayes factors from both groups we found that the model (with lower complexity) that did not include these receptors (our original model) outperformed the model with NMDA receptors on the Beta-Gamma data ranges. In contrast the Delta-optimised DCMs preferred the more complex model which included these NMDA receptor dynamics on spiny stellate cells.

Supplementary Figure 3

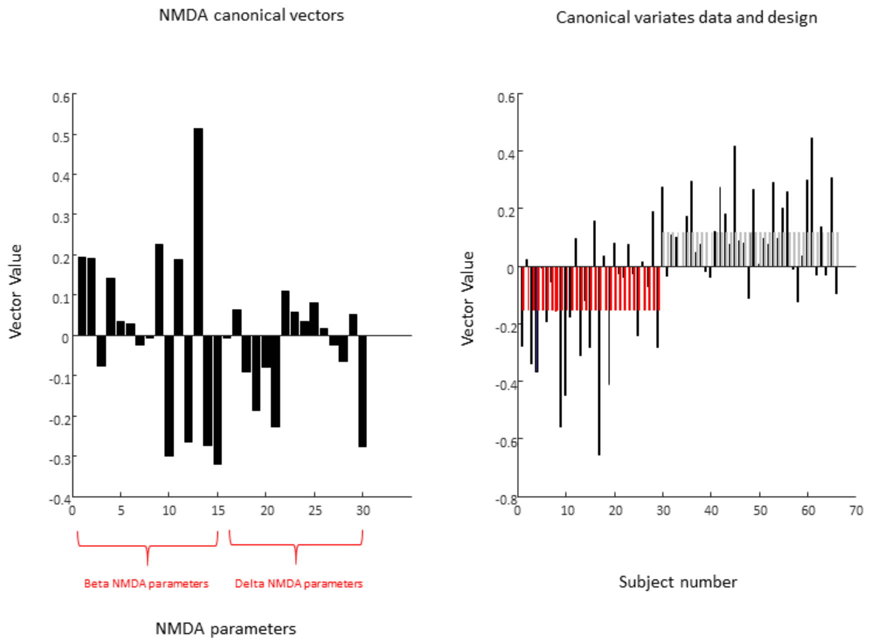

Supplementary Figure 3: **Canonical variate analysis**

Using a canonical variates analysis we mapped from a multivariate space (ion channel parameter estimates from the DCM to the canonical variate (of -1 for NMDAR antibody encephalitis or +1 other pathology). On the left we show the significant canonical vector coefficient for the 30 NMDA parameters (15 for the beta model and 15 for the delta model). On the right we show the true and predicted class (red: NMDAR antibody encephalitis; grey: other pathology). See Supplementary Table 4 for list of model parameters.

Supplementary Figure 4

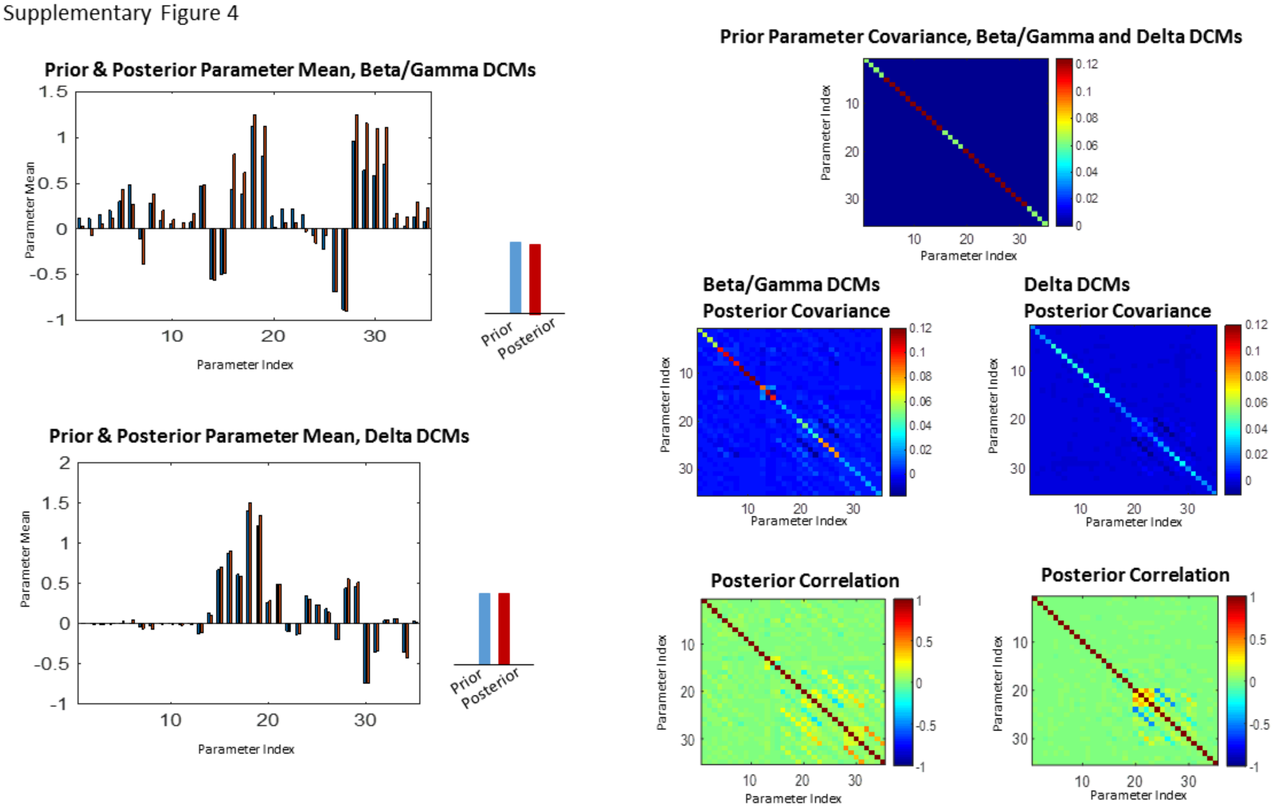

Supplementary Figure 4: **DCM Parameters: Priors & Posteriors**

On the left we plot the mean prior and posterior values (blue and red bars) for each of the key model parameters (listed in Table S4, along with posterior parameter ranges). On the right we show that a priori the parameters have zero covariation. Having fit the data we find the average covariance amongst parameters is low – when represented as the posterior correlation we find that on average the parameters show an absolute correlation of 0.03 and 0.02 for the beta-gamma and delta models respectively.

Supplementary Figure 5

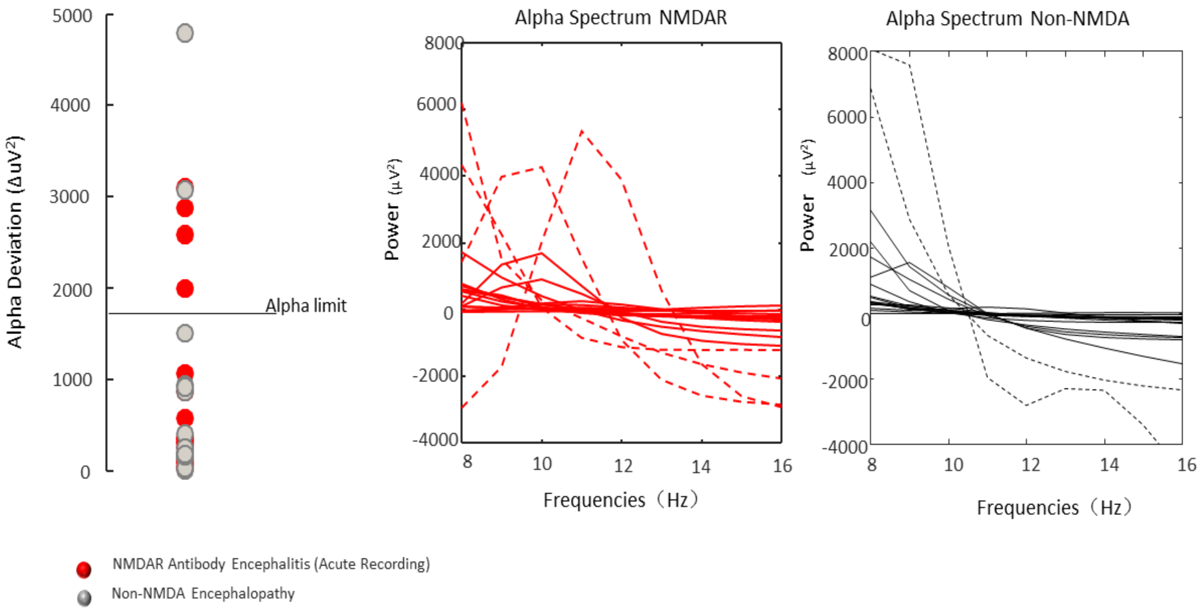

Supplementary Figure 5: Alpha power in patients with encephalopathy.

We determined that the 4 misclassifications in the canonical variate analysis for NMDA receptor antibody encephalitis patients could be attributed to high occipital alpha power in these patients. Thus, by accounting for this confound an accurate class label could be applied to all of our acute patients.

Supplementary Figure 6

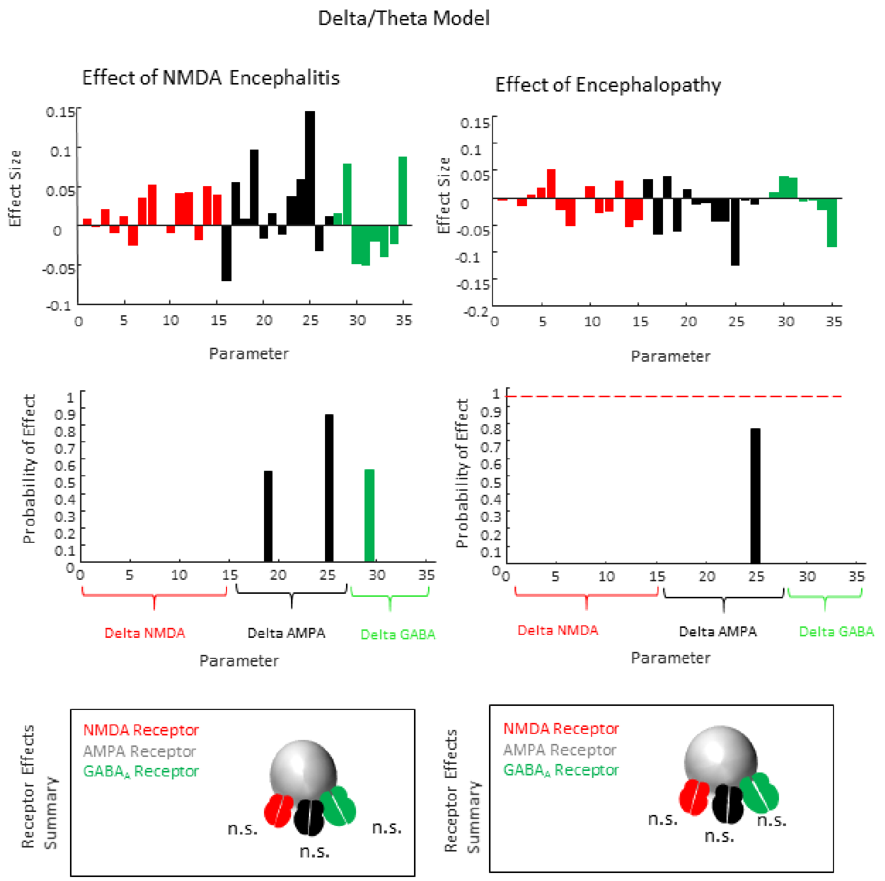

Supplementary Figure 6: Delta/Theta model

No significant parameter effects were observed for the second level regressors of NMDAR antibody encephalitis and encephalopathy using the delta/theta model. We speculate that this may result from the poorer model fits for these DCMs as compared to the beta/gamma models (Figure 2, main text). Here the panels describe the Bayesian covariate for each effect, the best reduced model parameter combination and the probability of a group effect in each parameter $p < 0.95$ in all cases.

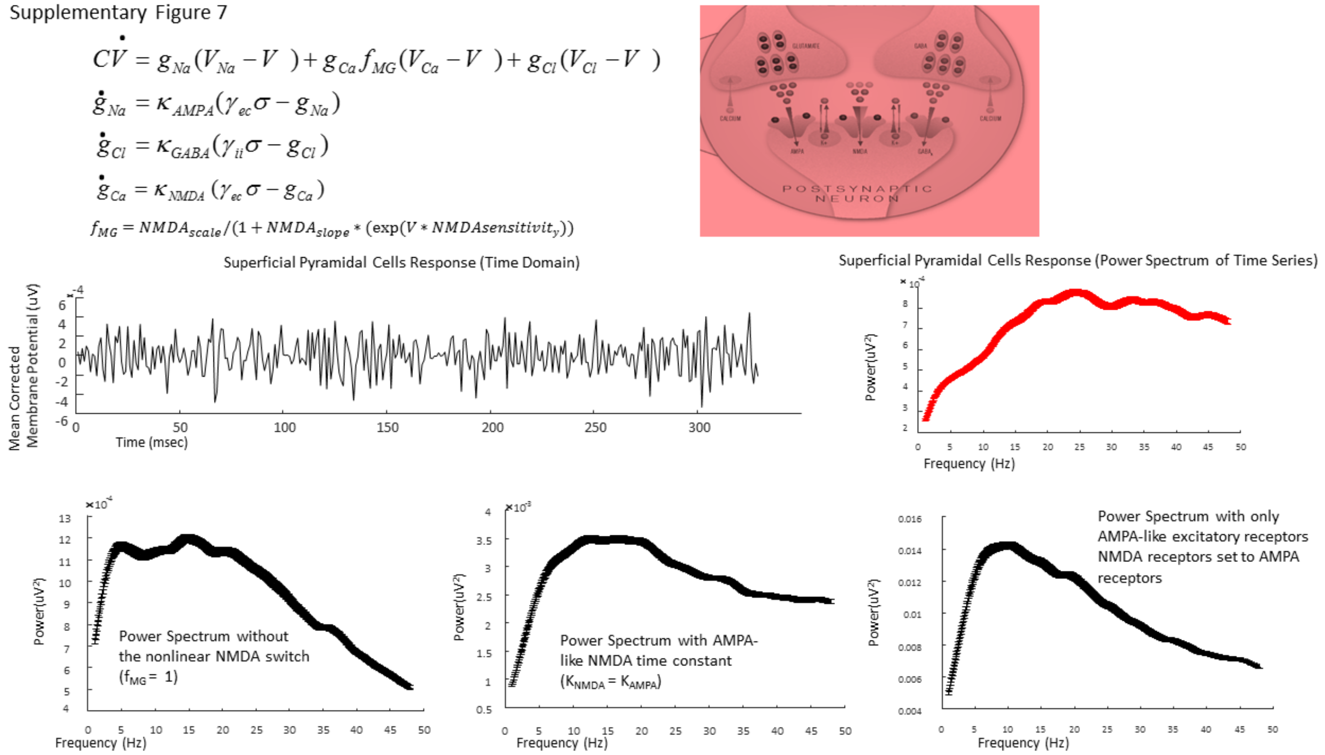

Supplementary Figure 7: Conductance based neural mass model & NMDA Receptor Effects

At each synaptic ensemble, membrane fluctuations in depolarisation (V) are modelled as dependent upon sodium, chloride, potassium and calcium ion channel conductances. Superficial and deep pyramidal cells and inhibitory interneurons are specified with three dynamic ion channel conductances (sodium, chloride and calcium, $g_{NA,}$ $g_{CA,}$ g_{Cl}); stellate cells are specified with two dynamic ion channel conductances (sodium and chloride), potassium operates at a constant leak level (g_K) (Gilbert et al. 2016). Coupling parameters link the cell populations within regions and between regions (e.g γ_{ec} links excitatory cells to the postsynaptic cells and γ_{ii} links inhibitory cells to the postsynaptic cells).

States & Parameters: C: membrane capactance; V – membrane voltage; V_x – channel specific reversal potential for ion; g_x – channel conductance; K_x – channel specific rate constant; γ_{ii} – connectivity (coupling) from inhibitory interneurons; γ_{ec} – connectivity (coupling) from pyramidal cells; σ – presynaptic firing rate; f_{MG} – non-linear magnesium block (f_{MG}); (Moran et

al. 2011). Parametrers for the sigmoid are from (Durstewitz, Seamans and Sejnowski 2000). Fixed values and parameter initialisations are detailed in the main text.

Lower panels: To investigate the precise role of NMDA receptor mechanisms on spectral outputs we simulated the 4-source network for a given set of posterior parameters (extracted from one subject's optimised parameters). Specifically, we simulated 100 instances of the time-domain response using randomised pink-noise responses over 330 msec. We simulated the optimised model dynamics then progressively degraded the NMDA receptor. Interestingly, the power spectrum showed high frequencies in the presence of the NMDA receptor, while removing the nonlinear magnesium blockade switch reduced some high-frequency power. Replacing the NMDA time constant with a faster AMPA like response resulted in a reduced high frequency response – but not to the same extent. Removing both resulted in strong low-frequency (alpha-like) activity. These simulations demonstrate that given the size of the state space (64) no one-to-one mapping from time constants exists, but that the NMDA receptor and the response it generates pervades throughout the spectrum. Lines denote mean over 100 simulations, presented with the standard error of the mean.

Supplementary Figure 8

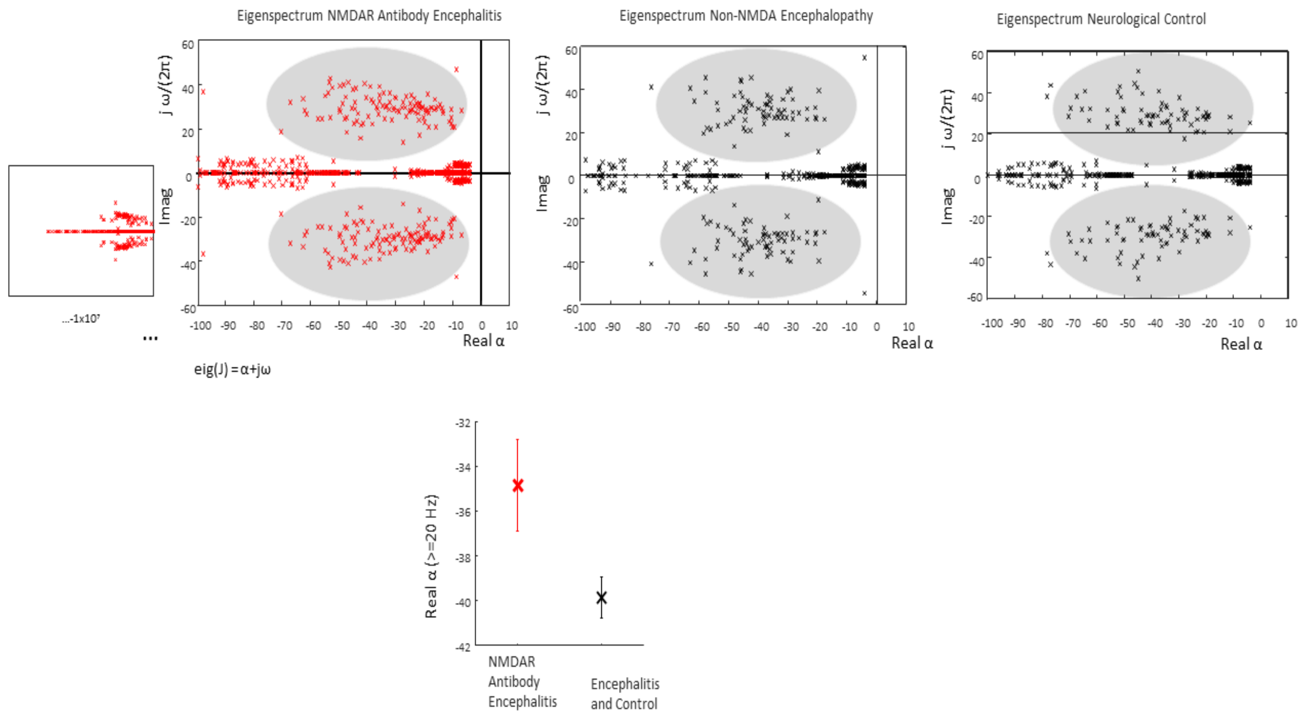

Supplementary Figure 8: Stability Analysis

We used the Jacobian based upon the optimised parameters from each individual to produce each individuals eigenspectrum (64 eigenvalues). Plotting these in the complex plan enables us to assess the stability properties of our cohort. We found that in the higher frequencies (>20 Hz), the anti-NMDA receptor encephalitis models tended to produce less stable dynamics (eigenvalues closer to the imaginary axis, shaded area). We illustrate just those eigenvalues from -100 to +10. No eigenvalues cross the imaginary axis. There was a significant difference between the real part of the NMDA-based eigenspectrum compared to the non NMDA encephalitis patients $p = 0.015$,unpaired student t test).

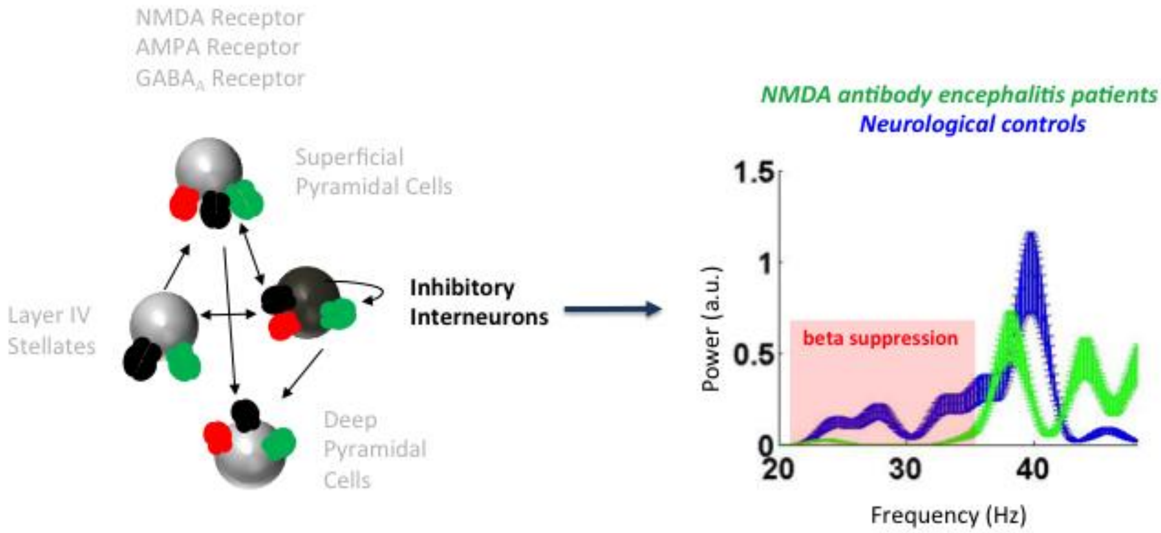

Supplementary Figure 9: Spectral response at inhibitory interneurons

To investigate the effect of our optimised parameter values on inhibitory cell firing, we simulated the spectral outputs from this cell layer in our network across patients, based on a Fourier transformation of the time-domain response using randomised pink-noise responses over 330 msec. The power spectrum showed significant suppression of beta frequencies in NMDA receptor antibody encephalitis patients compared to neurological controls. These simulations demonstrate that there is a significant inhibition of inhibitory cell firing in our model, arising from a non-linear mixture of parameter effects. Lines denote mean over patients, with the standard error of the mean.

Age	*Sex*	*Clinical details*	*Diagnosis*	*State during EEG*	*Medications*	*EEG features*
		Time since symptom onset		*Encephalopathy grade (0-4)*		*EEG encephalopathy grade (0-15)*
23	M	tremor, unsteadiness	NMDAR encephalitis	alert, anxious	nil	normal
		< 1 month		1		0
17	F	confusion, agitation, hemichorea-athetosis, dystonic posturing	NMDAR encephalitis	impaired concentration	nil	widespread 6Hz activity, poorly discernible PDR
		initially 4 years previously (current persistent cognitive symptoms)		1		4
56	M	confusion	NMDAR encephalitis	confused	PHY, VAL	normal, 8-9Hz PDR, moderate 5-7Hz theta
		<1 month		3		0
34	F	personality change, bursts of aggression, drowsiness, orofacial dyskinesia	NMDAR encephalitis	drowsy, withdrawn, agitated	Haloperidol, Olanzapine, PHY, LEV	normal, 10-11Hz PDR, small amplitude beta
		<1 month		2		0
19	M	intermittent unresponsiveness, confusion, drop attacks	NMDAR encephalitis	fluctuating consciousness	LEV	normal, 10Hz PDR, small amount of frontal slowing and beta
		<1 month		3		0
33	M	memory impairment, fatigue, cognitive slowing	NMDAR encephalitis	lethargic, mildly disorientated, slow speech	nil	9Hz PDR, minor amount of underlying posterior and temporal slow
		3 years		2		1
19	F	cognitive slowing, limb jerks, ataxia, hallucinations, psychosis, confusion, fatigue, memory difficulties, GTCS	NMDAR encephalitis	cognitive slowing, flat affect, mild disorientation	Clozapine, TOP, RET	high amplitude delta, occ. posterior spikes, intermittent 6Hz PDR
		2 years		1		9
16	M	confusion, altered personality, disinhibition, olfactory delusions, lethargy.	NMDAR encephalitis	confused, disorientated	nil	excess frontal delta, 10-11Hz PDR
		<1 month		3		1

30	M	altered behaviour, paranoia, apathy, disorientation, depression, suicidal ideation	NMDAR encephalitis	anxious, somnolent	Quetiapine, Sertraline	7Hz PDR, widespread 4-5Hz
		4 years (persistent mood and memory symptoms)		1		5
30	F	fluctuating consciousness	NMDAR encephalitis	unresponsive	PHY, VAL	bursts of slow waves, sharp theta transients and spikes
		<1 month		4		7
27	M	fluctuating behaviour, agitation, aggression	NMDAR encephalitis	agitated, altered affect	Clozapine	normal, 10Hz PDR
		7 years (initial encephalitic presentation; relapse <1 month with cognitive symptoms)		2		0
15	F	headache, confusion, personality change, psychosis, insomnia, withdrawal	NMDAR encephalitis	lethargic, poor concentration	Melatonin	9-10Hz PDR, intermittent theta-delta bi-frontal and temporal
		2 years		1		1
58	F	agitation, personality change	NMDAR encephalitis	awake, altered affect	Amitriptyline, OXC	normal, 8Hz PDR, mid temporal beta
		<1 month		2		0
30	F	drowsy unresponsive episodes, emotional lability, mutism, catatonia, auditory hallucinations, GTCS, focal motor seizures	NMDAR encephalitis	Severe cognitive slowing, lethargic, confused	LEV, Olanzapine	8-10Hz PDR, 1.5 - 7Hz generalised intermittent slowing
		6 months		3		6
19	F	Reduced consciousness, emotional lability, disinhibition, left arm posturing	NMDAR encephalitis	Awake but unresponsive	PHY	predominant 1-7Hz theta-delta
		<1 month		3		7
18	F	confusion, mutism, behavioural	NMDAR encephalitis	confused, impaired attention	CBZ	6-8Hz PDR,small amount of 2-4Hz

		change, dysphasia, amnesia				
		3 years		3		3
68	F	confusion, generalised seizures, aggression, hemiplegia	NMDAR encephalitis	alert, very mild disorientation	nil	normal, 9-10Hz PDR, single temporal spike
		3 months		1		0
78	M	tremor, myoclonic jerks, headache, collapse, gait apraxia	NMDAR encephalitis	mild drowsiness	Diazepam	6-7Hz PDR, excess 2-5Hz theta-delta
		11 months		1		3
57	F	jerky /choreiform movements, depression	NMDAR encephalitis	alert, mild impairment of awareness	nil	normal, indistinct PDR, low amplitude fast
		6 years (persistent cognitive symptoms)		1		0
17	F	personality change, anxiety, depression, focal seizures	NMDAR encephalitis	anxious, euphoria	CBZ	frontal intermittent rhythmic delta
		<1 month		2		1
23	F	behavioural change, confusion, dysphasia, psychosis, focal seizures, involuntary movements	NMDAR encephalitis	disorientated, incomprehensible speech	Clonazepam, Olanzapine	diffuse delta-theta and underlying low amplitude beta
		<1 month		3		9
26	F	fluctuating confusion, aphasia/mutism, unresponsive periods, rigidity, myoclonus, orofacial movements	NMDAR encephalitis	confused, aphasic, periods of unresponsiveness	Clonazepam, PHY, LEV, Lorazepam	fluctuating theta-delta activity
		<1 month		3		7
19	F	psychosis, confusion, emotional lability, collapse, catatonia, autonomic	NMDAR encephalitis	drowsy, emotionally labile, mild confusion	Lorazepam, Olanzapine	6-8Hz PDR, moderate theta-delta, excess beta

		dysfunction				
		<1 month		2		3
16	F	secondary generalised seizures, confusion, expressive dysphasia	NMDAR encephalitis	confused	LEV	9Hz PDR, left hemisphere long duration slow waves
		1 month		3		7
16	F	confusion, depression, agitation, mutism, leg and orofacial movements, GTCS	NMDAR encephalitis	confused	LEV, Lorazepam	diffuse 2.5 - 4Hz background, excess beta
		1 month		3		9
20	F	Psychosis with visual/auditory hallucinations, agitation, confusion, mutism, catatonia	NMDAR encephalitis	agitated, unresponsive, dysphasic	Lorazepam, Olanzapine	diffuse slow 1-3Hz anteriorly, excess beta posteriorly
		<1 month		3		9
59	F	headache, visual hallucinations	NMDAR encephalitis	sedated, unresponsive	Fentanyl, LEV, Propofol	theta-delta activity with right frontotemporal periodic discharge
		<1 month		4		9
26	M	psychosis, aggression, intermittent confusion	NMDAR encephalitis	alert, mild impairment of awareness	Lorazepam	normal, 11-12Hz PDR, moderate widespread beta
		<1 month		1		0
18	F	cognitive slowing	NMDAR encephalitis	slightly slow processing	LEV, VAL	left hemispheric theta-delta activity
		2 years, non-resolving symptoms		1		6

Supplementary Table 1: Clinical summary – NMDAR-antibody encephalitis patients

Clinical Summary of 29 patients diagnosed with NMDAR-antibody encephalitis, based on clinical presentation and positive serology for NMDA receptor antibodies. Main clinical features are summarized, with length of course prior to EEG, and clinical status at time of EEG. EEGs were available for 19 patients in the initial/acute phase of illness (3 months or less since initial presentation or relapse of clinical symptoms); other patients were in subacute or chronic stages of illness. Clinical severity of encephalopathy is graded using the West Haven criteria. A summary of EEG features and an EEG grade based upon the degree of encephalopathic features (see Supplementary Table 5) is also given. Medications with prominent CNS effects

(antiepileptics, sedatives, anxiolytics, antipsychotics and antidepressants) taken at the time of the EEG are also listed. Abbreviations: VAL (valproate), OXC (oxcarbazepine), CBZ (carbamazepine), CLO (clobazam), LTG (lamotrigine), LEV (levetiracetam), LAC (lacosamide), PHY (phenytoin), RET (retigabine), GTCS (generalized tonic-clonic seizure), PDR (posterior dominant rhythm)

Age	*Sex*	*Clinical details*	*Diagnosis*	*State during EEG* / *Encephalopathy grade (0-4)*	*Medications*	*EEG features* / *EEG encephalopathy grade (0-15)*
17	M	encephalopathy, seizures	mitochondrial encephalopathy (MERRF)	drowsy	nil	6-7Hz PDR, runs of delta
				1		7
24	F	unresponsive / comatose	subacute hydrocephalus	ventilated, unresponsive / minimally responsive	LEV, CBZ	unreactive delta-theta
				4		10
22	M	collapse/LOC, confusion	orbital cellulitis and septic encephalopathy	drowsy	nil	9-10Hz PDR, mild anterior delta
				1		1
38	M	collapse / LOC, hepatitis	drug-related encephalopathy	inappropriate behaviour, dysphasia	GBP, Opiates	8Hz PDR, 4-7Hz anterior theta
				1		4
22	F	unconscious post cardiac arrest	hypoxic ischaemic encephalopathy	sedated, ventilated, eye opening to stimuli	Lorazepam, Propofol	isoelectric periods, polymorphic slowing
				3		13
35	F	post status epilepticus, alcoholic liver disease	non-specific encephalopathy, no active seizures	unconscious, sedated, intubated, minimal response to stimuli	LEV, PHY, Propofol	theta-delta activity
				4		11
25	M	confusion	paraneoplastic (Ma2) limbic encephalitis	drowsy	LEV, PHY	intermittent 8-9Hz PDR, 5-6Hz frontally
				1		6
28	F	epilepsy, collapse with unresponsiveness, cerebral palsy	post-ictal encephalopathy	unresponsive to command, agitated	LEV	diffuse slow wave activity, 6-8Hz PDR with occasional theta-delta transients
				3		3
23	M	unresponsive episodes following head trauma	unspecified, post-traumatic	sedated, ventilated, unresponsive	Amitriptyline, Fentanyl, Propofol, LEV	high amplitude delta, runs of central theta
				4		9
35	M	encephalopathic, previous non-convulsive status epilepticus	unspecified, post-ictal state	unresponsive	CBZ , ZON	8-14Hz PDR, delta anteriorly, excess fast
				4		9
37	F	collapse/LOC following air embolus, ischaemic hepatitis	hypoxic ischaemic encephalopathy	spontaneous eye opening, unable to follow commands	Fentanyl	low amplitude, mixed polymorphic 1-8Hz
				3		12
22	M	unconscious following subarachnoid haemorrhage	drug-related encephalopathy	sedated, unresponsive	Fentanyl, Midazolam, Propofol,	high amplitude delta, intermittent burst suppression
				4		7
26	F	subacute	cerebral	confused	nil	4-7Hz PDR, runs of

		confusion, hearing loss	vasculopathy (Susac's syndrome)			frontotemporal delta
				3		7
35	F	episodic confusion / altered behaviour	probable drug-related (opiate) encephalopathy	mild disorientation	LTG, Opiates, Amitriptyline, Fluoxetine	8-9Hz PDR, frontotemporal 3-5Hz
				1		7
47	F	traumatic subarachnoid and intracerebral haemorrhage	post-traumatic encephalopathy	sedated, unresponsive	PHY, Propofol, Fentanyl, Midazolam	5-7Hz PDR, low amplitude
				4		9
32	M	behavioural change, vacant episodes. Focal epilepsy, cerebral palsy	unspecified encephalopathy	mild disorientation	LEV, PHY, CLO, TOP, Baclofen	intermittent 5-7Hz, runs of central and frontal 2-4Hz
				1		7
32	M	unconscious following spontaneous subarachnoid haemorrhage	post-haemorrhagic encephalopathy	sedated, unresponsive	LEV, PHY, Fentanyl, Propofol	1-2Hz delta, frontocentral 7Hz theta
				4		10
23	M	auditory hallucinations, behavioural change	autoimmune (anti-glycine receptor) encephalitis	drowsy	VAL, Olanzapine	5-6Hz PDR, frontal delta
				1		4

Supplementary Table 2: Clinical summary – non-NMDA encephalopathy patients

Clinical Summary of 18 patients with acute encephalopathies, from a variety of metabolic, toxic, inflammatory and structural causes. Main clinical features are summarized, with clinical status at time of EEG and clinical severity of encephalopathy graded using the West Haven criteria. A summary of EEG features and an EEG grade based upon the degree of encephalopathic features (see Supplementary Table 5) is also given. Medications with prominent CNS effects (including antiepileptics, anaesthetics, anxiolytics, antipsychotics, analgesics and antidepressants) taken at the time of the EEG are also listed. Abbreviations: VAL (valproate), CBZ (carbamazepine), OXC (oxcarbazepine), CLO (clobazam), LTG (lamotrigine), LEV (levetiracetam), LAC (lacosamide), PHY (phenytoin) ZON (zonisamide), TOP (topiramate). GTCS (generalized tonic clonic seizure). PDR (posterior dominant rhythm). MERRF (mitochondrial epilepsy with ragged red fibres.

Age	*Sex*	*Clinical details*	*Diagnosis*	*State during EEG*	*Medications*	*EEG features*
20	F	collapse/LOC	syncope	alert	nil	9-10Hz PDR, temporal theta
24	F	collapse/LOC	probable syncope	alert	nil	10Hz PDR
32	F	altered consciousness and amnesia	focal onset seizure (temporal lobe epilepsy)	alert	nil	10Hz PDR, sharp temporal transients
20	M	behavioural change, autism	unspecified, no evidence of epilepsy	alert	nil	9-10Hz PDR, frontal theta
18	F	collapse/LOC	probable syncope	alert	nil	10Hz PDR
25	F	collapse/LOC	possible isolated seizure	alert	nil	10Hz PDR
18	F	altered behaviour, low mood	unspecified, no evidence of epilepsy	alert	nil	11Hz PDR
31	F	limb jerks	probable psychogenic movement disorder	alert	nil	9Hz PDR
23	M	LOC with head version	focal onset seizure	alert	nil	9Hz PDR
20	M	LOC	primary generalised epilepsy	alert	nil	8-10Hz PDR, single generalised poly-spike wave discharge
23	F	LOC with visual aura	unspecified, no clear evidence of epilepsy	alert	VAL	9-10Hz PDR
32	F	nocturnal generalised seizures	primary generalised epilepsy	alert	CLO, OXC	9-10Hz PDR, frontal theta
17	F	nocturnal generalised seizures	primary generalised epilepsy with photosensitivity	alert	LTG	10Hz PDR
22	F	cephalic and autonomic aura, LOC	probable focal onset seizure	alert	nil	11-12Hz PDR, mid temporal theta
18	F	limb jerks	non-epileptic myoclonus	alert	Citalopram, Codeine	10-11Hz PDR, central theta
36	M	generalised tonic-clonic seizure	alcohol withdrawal seizure	alert	Mirtazepam	9-10Hz PDR, frontocentral theta-delta
39	F	collapse/LOC	probable syncope	alert	nil	9Hz PDR
38	M	limb paraesthesiae, LOC	focal onset seizure	alert	LEV, Methadone	7-8Hz PDR, frontal theta-delta

Supplementary Table 3: Clinical summary – non-encephalopathic patients

Clinical Summary of 18 non-encephalopathic neurological patients. Main clinical features are summarized, including clinical status at time of EEG. A summary of EEG features is also given. Medications with prominent CNS effects (antiepileptics, sedatives, anxiolytics, antipsychotics and antidepressants) taken at the time of the EEG are also listed. Abbreviations: VAL (valproate), OXC (oxcarbazepine), CLO (clobazam), LTG (lamotrigine), LEV (levetiracetam),

LAC (lacosamide), PHY (phenytoin). GTCS (generalized tonic clonic seizure). PDR (posterior dominant rhythm).

Parameter Number	Description	DCM Posterior parameter values Beta (20-48Hz)		Delta (1-4Hz)	
NMDA Parameters		Min	Max	Min	Max
1	RATE OF NMDA CHANNEL OPENING LEFT PARIETAL CORTEX (LPC)	0.448	1.5306	0.8122	1.2039
2	RATE OF NMDA CHANNEL OPENING RIGHT PARIETAL CORTEX (RPC)	0.2843	1.478	0.6164	1.0265
3	RATE OF NMDA CHANNEL OPENING LEFT PREFRONTAL CORTEX (LPFC)	0.4015	4.3075	0.8855	1.622
4	RATE OF NMDA CHANNEL OPENING RIGHT PREFRONTAL CORTEX (RPFC)	0.423	4.7258	0.9099	1.9547
5	INTRINSIC CONNECTIONS TO NMDA RECEPTORS (EXCITATORY CELLS (LPC)	0.4253	3.4478	0.9632	2.5559
6	INTRINSIC CONNECTIONS TO NMDA RECEPTORS (EXCITATORY CELLS (RPC)	0.5283	2.6944	0.9695	2.1356
7	INTRINSIC CONNECTIONS TO NMDA RECEPTORS (EXCITATORY CELLS (LPFC)	0.1496	2.4817	0.5322	1.0834
8	INTRINSIC CONNECTIONS TO NMDA RECEPTORS (EXCITATORY CELLS (RPFC)	0.3735	4.8616	0.2917	1.2138
9	INTRINSIC CONNECTIONS TO NMDA RECEPTORS (INHIBITORY CELLS (LPC)	0.7744	3.8477	0.8284	1.0506
10	INTRINSIC CONNECTIONS TO NMDA RECEPTORS (INHIBITORY CELLS (RPC)	0.6256	4.7342	0.6332	1.221
11	INTRINSIC CONNECTIONS TO NMDA RECEPTORS (INHIBITORY CELLS (LPFC)	0.4921	4.3832	0.6269	1.6281
12	INTRINSIC CONNECTIONS TO NMDA RECEPTORS (INHIBITORY CELLS (RPFC)	0.8832	2.186	0.7145	1.28
13	MAGNESIUM BLOCK VOLTAGE GATE AMPLITUDE	1.0435	2.0977	0.745	1.687
14	MAGNESIUM BLOCK VOLTAGE GATE SLOPE	0.3382	0.9127	0.5895	1.3298
15	MAGNESIUM BLOCK VOLTAGE GATE VOLTAGE SENSITIVITY	0.351	1.2881	1.3187	4.466
AMPA Parameters		Min	Max	Min	Max
16	RATE OF AMPA CHANNEL OPENING LPC	1.1047	6.3948	0.9435	4.8359
17	RATE OF AMPA CHANNEL OPENING RPC	0.6603	7.2411	1.034	2.7401
18	RATE OF AMPA CHANNEL OPENING LPFC	1.9646	5.1669	2.5698	11.2285
19	RATE OF AMPA CHANNEL OPENING RPFC	1.3837	5.5957	2.8258	8.3446
20	INTRINSIC CONNECTIONS TO AMPA RECEPTORS LPC	0.2439	1.3058	1.2013	2.1775
21	INTRINSIC CONNECTIONS TO AMPA RECEPTORS RPC	0.5486	1.3324	1.2439	1.9171
22	INTRINSIC CONNECTIONS TO AMPA RECEPTORS LPFC	0.4747	1.4634	0.8304	1.096
23	INTRINSIC CONNECTIONS TO AMPA RECEPTORS RPFC	0.3707	1.3728	0.7715	1.0049
24	EXTRINSIC CONNECTION LPC TO LPFC	0.1712	1.3405	0.831	1.9753
25	EXTRINSIC CONNECTION RPC TO RPFC	0.2147	1.9205	0.6799	2.4789
26	EXTRINSIC CONNECTION LPFC TO LPC	0.318	1.16	0.5732	1.2997
27	EXTRINSIC CONNECTION RPFC TO RPC	0.2097	0.902	0.7134	1.0634
GABA Parameters		Min	Max	Min	Max
28	INTRINSIC CONNECTIONS TO GABA RECEPTORS LPC	2.3473	6.5402	0.8576	5.188
29	INTRINSIC CONNECTIONS TO GABA RECEPTORS RPC	1.6968	6.482	0.7927	12.9927
30	INTRINSIC CONNECTIONS TO GABA RECEPTORS LPFC	0.432	5.3873	0.2922	0.7394
31	INTRINSIC CONNECTIONS TO GABA RECEPTORS RPFC	1.0399	8.7701	0.455	1.0886
32	RATE OF GABA CHANNEL OPENING LPC	0.5873	6.5809	0.825	1.2114
33	RATE OF GABA CHANNEL OPENING RPC	0.6503	3.626	0.3819	1.817
34	RATE OF GABA CHANNEL OPENING LPFC	0.8206	5.4654	0.1998	1.1091
35	RATE OF GABA CHANNEL OPENING RPFC	0.4655	3.6615	0.3776	1.4603

Supplementary Table 4: Parameter list for biophysical models.

NMDA, AMPA and GABA receptor model parameters, posterior parameter ranges.

0	normal
1	normal background with intermittent slowing
2	slow background 7-8Hz without theta-delta waves
3	slow background 7-8Hz with theta-delta waves
4	4-6Hz background without theta-delta waves
5	4-6Hz background with theta-delta waves
6	dominant theta-delta with normal background activity
7	dominant theta-delta with slow background activity
8	dominant delta with normal background activity
9	dominant delta with slow background activity
10	moderate to high amplitude delta >50uV with no background reactivity
11	low amplitude <50uV delta with no background reactivity
12	burst suppression with suppression period <5s
13	burst suppression with suppression period >5s
14	near electrocerebral silence
15	electrocerebral silence

Supplementary Table 5: Grading scales for electrographic and clinical features of encephalopathy

15 point grading scale: electrographic features of encephalopathy correlating with low to high severity.

Supplementary Methods

To estimate model parameters, θ, DCM employs a variational Bayesian approach using a mean field partition to iteratively update a multivariate Gaussian over model parameters and a gaussian hyperparameter that models the level of additive measurement noise, γ. This procedure minimizes a precision weighted square error term and a complexity parameter – the cost function is known as the Free Energy, *F* given the complex spectral energy, *y* and the model prediction *h*(μ). The complexity penalty is the Kullback Leibler divergence between the prior $N\sim(\mu_\theta, C_\theta)$and posterior densities, $N\sim\left(\mu_{\theta|y}, C_{\theta|y}\right)$, penalizing more independence amongst the parameters, dependencies amongst posterior parameters and deviations in the posterior mean from the prior mean.

$$F = \frac{1}{2}(y-h)'(y-h) + \frac{1}{2}\ln|C_\theta| - \frac{1}{2}\ln\left|C_{\theta|y}\right| + \frac{1}{2}(\mu_{\theta|y} - \mu_\theta)'C_\theta^{-1}(\mu_{\theta|y} - \mu_\theta)$$

Following Friston et al., (2007) one performs an E-step of gradient descent on the free energy to optimise the posterior moments. The M-step performs a descent on the free energy to update the hyperparameter. This is repeated until convergence where the objective function *F* changes by less than 10^{-2}.

Pseudo code, see (Friston et al. 2007):

E-step

$$C_{\theta|y}^{-1} = h'C_{\theta|y}^{-1}h + C_\theta^{-1}$$
$$\Delta\mu_{\theta|y} = -C_{\theta|y}^{-1}(h'C_{\theta|y}^{-1}(y-h) + C_\theta^{-1}(\mu_{\theta|y} - \mu_\theta))$$

M-step

$$I = \frac{1}{2}tr\left(P\left((y-h)'(y-h) - \text{ Id} * \gamma + hC_{\theta|y}h\right)\right)$$
$$P = \frac{\delta C_{\theta|y}^{-1}}{\delta\gamma}$$
$$\Delta\gamma = -(\frac{\delta I}{\delta\gamma})^{-1}I$$

We plot the prior and posterior means and covariances in figure S3. From the posterior correlation matrices (a normalized covariance matrix to reveal parameter dependencies) we find that the mean absolute correlation is 0.03 for the beta/gamma models and 0.02 for the delta models. Hence, we can conclude that our key parameters contribute differentially to the signal and are locally identifiable. However, given the presence of local minima in these models, the identifiability is local in the neighbourhood of *F* (See definition 2 in (Ljung and Glad 1994)).

References

Durstewitz, D., J. K. Seamans & T. J. Sejnowski (2000) Dopamine-mediated stabilization of delay-period activity in a network model of prefrontal cortex. *Journal of neurophysiology,* 83**,** 1733-1750.

Friston, K., J. Mattout, N. Trujillo-Barreto, J. Ashburner & W. Penny (2007) Variational free energy and the Laplace approximation. *Neuroimage,* 34**,** 220-234.

Gilbert, J. R., M. Symmonds, M. G. Hanna, R. J. Dolan, K. J. Friston & R. J. Moran (2016) Profiling neuronal ion channelopathies with non-invasive brain imaging and dynamic causal models: case studies of single gene mutations. *Neuroimage,* 124**,** 43-53.

Ljung, L. & T. Glad (1994) On global identifiability for arbitrary model parametrizations. *Automatica,* 30**,** 265-276.

Moran, R. J., K. E. Stephan, R. J. Dolan & K. J. Friston (2011) Consistent spectral predictors for dynamic causal models of steady-state responses. *Neuroimage,* 55**,** 1694-1708.
